# A Theory of Change for Web-Based Therapy and Support Services for Children and Young People: Collaborative Qualitative Exploration

**DOI:** 10.2196/23193

**Published:** 2021-03-22

**Authors:** Terry Hanley, Aaron Sefi, Janet Grauberg, Julie Prescott, Andre Etchebarne

**Affiliations:** 1 University of Manchester Manchester United Kingdom; 2 Kooth London United Kingdom; 3 University of Bolton Bolton United Kingdom

**Keywords:** telepsychology, digital mental health, online therapy, young people, Kooth, Theory of Change, positive virtual ecosystems

## Abstract

**Background:**

Web-based counseling and support has become increasingly commonplace for children and young people (CYP). Currently, there is limited research that focuses on the mechanisms of change within complex telepsychology platforms, a factor that makes designing and implementing outcome measures challenging.

**Objective:**

This project aims to articulate a theory of change (ToC) for Kooth, a web-based therapy and support platform for CYP.

**Methods:**

A collaborative qualitative research design involving professional staff, academic partners, and young people was used to develop the ToC. The following three major reflective phases were engaged: a scoping workshop involving professional staff and academic partners, a series of explorative projects were completed to inform the development of the ToC, and the draft ToC was reviewed for coherence by key stakeholders (young people, online professionals, and service managers).

**Results:**

A collaboratively developed ToC was presented. This was divided into the conditions that lead to individuals wanting to access web-based therapy and support (eg, individuals wanting support there and then or quickly), the mode of service delivery (eg, skilled and experienced professionals able to build empathetic relationships with CYP), and the observed and reported changes that occur as a consequence of using the service (eg, individuals being better able to manage current and future situations).

**Conclusions:**

Developing the ToC helps to shed light on how web-based therapy and support services aid the mental health and well-being of CYP. Furthermore, it helps to understand the development of *positive virtual ecosystems* and can be used to devise evaluative tools for CYP telepsychology providers.

## Introduction

This paper reports a study conducted as a collaboration between professionals working for Kooth and academic researchers working within the United Kingdom. Kooth is a web-based counseling and support service for young people and young adults (aged 11-25 years). It is anonymous at the point of access, with young users typically finding out about the service through educational providers and only having to provide limited information on registration. In 2019, it received approximately 1700 log-ins per day, a figure that increased to just over 3000 log-ins on the day in which the COVID-19 pandemic caused schools to be shut down and the government to restrict movement of the UK population (March 20, 2020). Of these log-ins, just under 1000 were a result of new registrations, which represented an increase of over 50% in the trend for registrations earlier in that week.

During the evolution of the Kooth service, it became clear that existing tools for measuring outcomes did not prove fit for purpose, with both the format of the tools and the concepts that they measure proving to be problematic [1]. In this regard, further scrutiny of the work that was undertaken on the website proved to be necessary. As a first step, it was considered necessary to review the work of the service as a whole and develop a theory of change (ToC) so as to consider what might be the most appropriate means of evaluating the work that the service engages in.

### Reflexive Statement by the Authors

Before continuing, it is important to note that most of the team members contributing to this paper have been involved in the development of web-based therapeutic services for several years. As such, they view developments in this arena as both necessary and inevitable. Three of the team members have actively researched web-based therapeutic provision (TH, JP, and AS), and 2 of the team members have been involved in the development of such services (TH and AS). One team member provides consultancy related to the development of theories of change (JG), and one team member is a trainee counseling psychologist with an interest in developing accessible therapeutic services (AE).

### Therapeutic Provision for Children and Young People

There is a global acknowledgment that children and young people (CYP) would benefit from additional support to improve their mental health and well-being [2,3]. In the United Kingdom, where this study has been completed, research indicates that as many as 1 in 8 young people will experience mental health issues between the ages of 5 and 19 years [4]. Furthermore, it is believed that a large number of young people belonging to this group will continue to experience mental health difficulties as they become adults, thus impacting wider issues such as individuals’ future employment [3]. Although there is evidence to suggest that CYP can benefit from therapeutic interventions provided by a wide variety of services [5], access to support varies greatly depending on the geographical location.

In the United Kingdom, there is a wide range of ways in which young people might access psychological support. These include statutory health services in the form of child and adolescent mental health services (CAMHS), school-based counseling and psychology services, community-based services, and mediated services (web-based and telephone) [6-8]. Recent statistics indicate that the average wait time for statutory support is 56 days, with the shortest time being 49 days and the longest being 65 days [9]. Furthermore, the waiting times assume that young people will have their referral accepted to CAMHS. Owing to the emphasis on meeting specific service criteria, approximately three-quarters of young people with a diagnosable mental health condition will have their referral rejected. Overall, this is reflective of the disparities between adult and young people’s mental health provision, with it being estimated that, despite making up 20% of the population, only 10% of the mental health budget is spent on CYP [10]. The limited resources available are also the main reason that additional NGOs have developed services to fill the gaps within this provision.

### Young People, Mental Health and Well-being, and the Internet

Young people have been described as *digital natives* [11], notably individuals who have never lived in a world without the internet. Consequently, within countries that have widespread access to the internet, young people and young adults are now viewed as the biggest users of the internet and social media [12]. Furthermore, research examining help-seeking behaviors of this group suggests that they use the internet as their first point of call for support for issues related to mental health and well-being [13,14]. The type of support available on the web includes informational support (eg, websites that include information about particular issues) and emotional support (eg, social connections and connections with professionals) [15,16]. More specifically, this can include websites that include (1) informative content, (2) web-based question and answer sessions, (3) online forums [16], (4) stand-alone therapeutic programs or apps [17], and (5) web-based contact with professionals (eg, web-based therapists) [18].

Kooth, the service that this project has worked alongside, is one such service that offers a suite of web-based support options to CYP. It is a free web-based therapy and support service that supports over half a million CYP in the United Kingdom primarily through text-based support [19]. It has grown swiftly during this period and, at the time of writing, is funded by 115 of the 135 National Health Service Clinical Commissioning Groups in England. It differs from many web-based services, as it aims to explicitly integrate itself into existing local services and works alongside community-based professionals such as teachers, doctors, psychologists, and community health teams. The CYP who access the service remain anonymous and can tailor the support they receive, with individuals choosing between support that involves direct contact with professionals or not. For instance, some individuals may only read web-based content in the form of psychoeducational articles or online forums. Others may directly communicate with professionals by using synchronous and asynchronous chat options. This decision is led by the individuals accessing the service rather than the professionals offering support, and many individuals choose to use a combination of the above. This is in keeping with the organization’s humanistic value base [20,21], an underpinning that is positively focused and prizes the agency of the individuals seeking support [22,23], and the professionals who offer support adopt a pluralistic goal-directed therapeutic approach [24,25]. These professionals include counselors, psychologists, psychotherapists, and social workers, and they work closely alongside those with more specific remits to write content and manage the technical side of the website.

The provision of anonymous support to CYP is a contentious arena. For some, the delivery of such services can be viewed as risky or dangerous. This proves particularly the case where an individual may be at risk of serious harm to themselves or another person, with some countries insisting that such support can only be provided with the consent of parents or caregivers. However, in the United Kingdom, there is a long tradition of providing anonymous telephone support, with ChildLine, an anonymous telephone helpline for CYP, which was set up in 1986 following a public campaign focusing on the cruelty and abuse affecting CYP [26]. This service purposefully offered anonymity to its users with a view to provide the much-needed support that would most likely not have been accessed if only offered face-to-face. A number of web-based services now work in this way and the young people who access these services often highlight the importance of being able to access support anonymously [27]. Individuals who access such services do so with a wide range of complex needs [1] and typically these anonymous services work with individuals regardless of the issue disclosed. Risk is not ignored; however, professionals will support individuals to access additional services if needed. In doing so, they will attempt to work at the pace of the person obtaining support and not remove support if individuals choose not to provide further identifiable information.

### Measuring Outcomes in Web-Based Therapy and Support Services

The current climate of mental health provision requires service providers to demonstrate the benefits of their work to those commissioning them. Commonly, these take the form of aggregated scores collated from self-report outcome measures reflecting upon the reduction of negative symptoms or the success of goals. There are numerous accepted processes and protocols for doing so, with organizations such as the Child Outcomes Research Consortium, creating specific guidance and recommendations. However, such measures have proven to be difficult to transfer into virtual environments. Difficulties have included practical issues such as transferring measures into web-based formats and navigating copyright issues and, more significantly, considering whether the measure itself is used in the same way as it would be in face-to-face relationships [1]. Therefore, there are suggestions that individuals accessing web-based services, particularly anonymous web-based services, do so for different reasons than those accessing face-to-face therapy. Indeed, exploration around the nature of web-based therapeutic relationships with CYP suggests that specific web-based issues, such as the safety afforded by the anonymous relationship, help to enhance the work entered into [27,28]. As a consequence of these differences, it can be argued that it is necessary to develop evaluation tools that take into account the complex environment more fully and make the best use of the technology available to ensure that any tools that are adopted are user friendly.

### Developing a ToC

A ToC is an outcomes-based approach that can be used to identify and evaluate how programs and services achieve their stated goals of change. ToC has been defined as “the description of a sequence of events that is expected to lead to a particular desired outcome” [29]. Previous reviews indicate that ToCs should be flexible, be reflective, and ensure that any assumptions are explicitly stated [30]. When designing a ToC, Kail and Lumley [31] identified the following 5-step process:

1. Identify a realistic and definite goal.
2. Work backward from the goal to work out the intermediate outcomes.
3. Establish links between outcomes and their order by working out causes and effects.
4. Work out which activities lead to which outcomes.
5. Identify what else is needed for the intervention to work.

Developing a ToC can have several benefits for key stakeholders in a service. Staff members typically play an active role in the development of the ToC, owing to their perspective on what works are valued. This collaborative process can help to identify hidden perspectives while also helping to motivate staff by showing them how their contributions fit into the service-level goals [32]. In addition, a ToC clarifies what needs to be measured, as the assumptions about what makes the intervention work are identified. A ToC can also be used as a heuristic to help guide the creation of ToC for similar programs [33]. For example, this could be for applying the lessons learned from a ToC for a web-based CYP service to the development of ToC for a web-based service for adults. Finally, there is the added benefit that a ToC can be both *retrospective* by evaluating the efficacy of a service to date and *prospective* when used as a tool to support the planning of service development [34,35]. ToCs have been used in a range of contexts for young people, including sports programs within the youth justice service [34], school-based interventions for students and their families [36], and well-being programs for those unable to access mainstream education [37].

### Aims, Rationale, and Research Questions

Given the complex nature of the web-based therapeutic environment, the aims of this study are to develop a ToC and map out the ways in which CYP access support on the Kooth platform. As such, the research question for this project is “what do key stakeholders identify as the core elements of a ToC for an anonymous online therapy and support service?”

## Methods

### Design

Working *with* organizations, instead of examining them from afar, is advocated to create more ecologically relevant pieces of research [38,39]. This project therefore reflects upon a collaborative piece of research between professionals from the Kooth service and academics with an interest in web-based therapeutic resources and theories of change. The close working between researchers and professional staff members was purposefully egalitarian in nature so as not to prize one set of knowledge or interpretation over the other. As such, the professionals involved in the project were both coresearchers [40] and practitioner researchers [41] responsible for constructing the ideas presented in this paper.

In keeping with the exploratory perspective adopted, the project has a social constructionist epistemology [42] and proved to be primarily inductive in nature [43]. However, it was acknowledged that this blank canvas approach was influenced by the theoretical perspectives of the individuals involved. For instance, as noted in the background of this paper, the principles of humanistic psychology were greatly valued by both the service and the researchers involved. As such, a critically reflexive approach to the research was adopted for the conceptual presentations [44,45]. In practical terms, the researchers have purposefully engaged in critical discussions and sought external dialog to inform the development of the proposed theory. Here, it is noteworthy that ideas from good practice guidelines for qualitative research that advocate coherence checks with a variety of partners [46,47] have been used to enhance the overall trustworthiness of the synthesis that is presented.

As described briefly in the background section of this paper, the study made use of ToC methodologies to help devise a deeper understanding of the work that the Kooth service enters with CYP. In particular, the ToC methodology focused on gaining insights into the specific conditions that lead individuals to use the service in question, the mode of delivery that services are offered, and the change people report or observe as a consequence of using the service [31]. These methods are purposefully collaborative in their approach and are primarily inductive at the starting point of the project.

The following sections outline this collaborative approach. Initially, the three phases of the project are briefly described: (1) a scoping workshop activity is described; this is followed by (2) the facilitation of a series of practitioner researcher activities and (3) a final coherence checking process. Each phase refers to the individuals involved, the process of generating data, and the data analysis procedures that are engaged.

### Phase 1: Scoping Workshop

**Figure 1:**
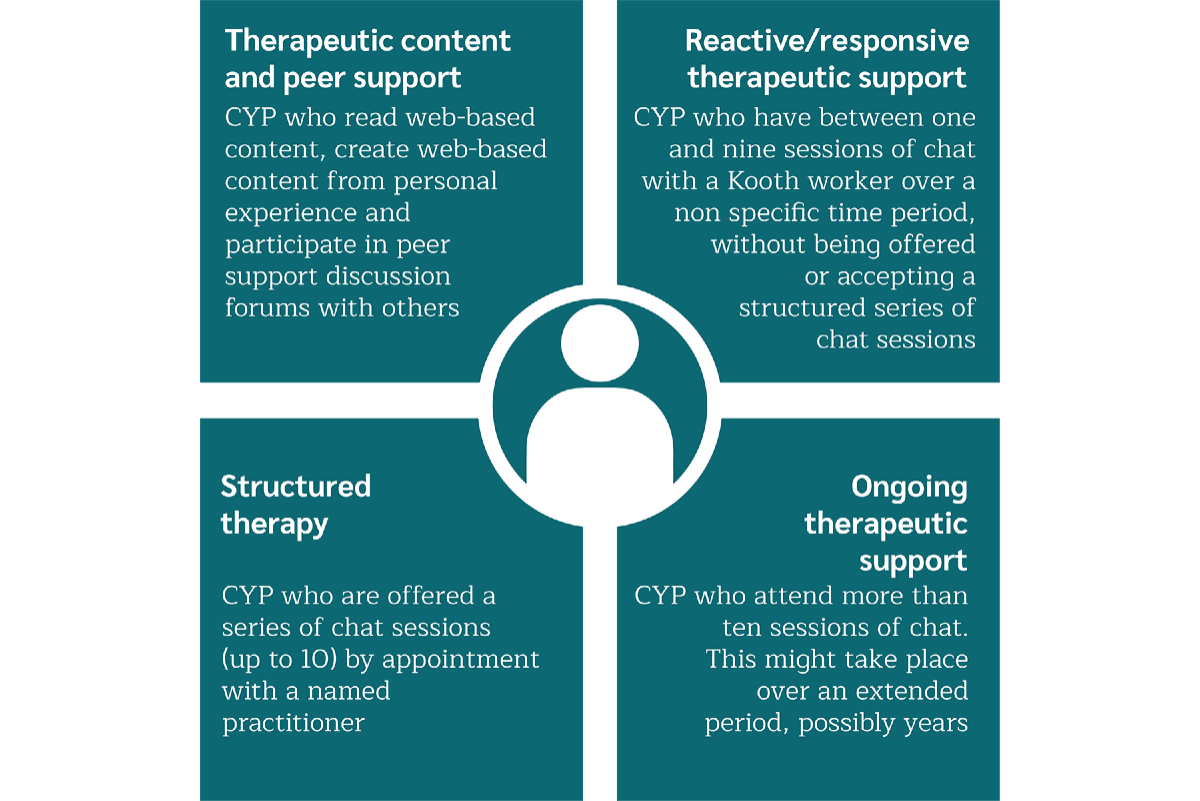
Descriptions of the 4 service pathways for Kooth. CYP: children and young people.

A workshop was held by combining 11 Kooth staff (5 therapists, 2 emotional well-being practitioners, 1 community engagement worker, 1 learning and development coordinator, and 2 research staff) and academic partners (TH and JG). As the staff members possess intimate knowledge regarding the way CYP make use of and benefit from the service, this workshop proved to be a vital starting point to direct the project. During the workshop, a series of presentations were given to provide information about the purpose of the project and the notion of a ToC. Focused conversations [48] were then held around the different ways in which CYP engaged with the Kooth service. These focused on the following 3 aspects: (1) the way in which CYP use the service (specifically highlighting any conditions present that mean CYP wish to use a web-based therapy and support service); (2) the ways in which CYP engage with the service (modes of delivery and the activities of key players); and (3) the impacts observed or reported while using the service (identified outputs and observed change). At the end of the workshop, seeds were sown about the ToC itself, with a rough draft being presented, and 4 support pathways were identified that represent the way in which individuals use the Kooth service. Figure 1 provides a description of these pathways.

### Phase 2: Practitioner Researcher Pathway Explorations

Following the initial scoping workshop, 6 practitioners (4 therapists, 1 emotional well-being practitioner, and 1 community engagement worker) working for Kooth were invited to become practitioner researchers [41]. These individuals worked in groups to develop an understanding of the four agreed service pathways previously noted. The practitioner researchers involved were invited based on their experience and prior training in research methods, and their understanding of the pathways employed in the Kooth service.

The practitioner researcher teams were assigned to specific pathways and instructed to explore these in detail to develop an understanding of the ToC for that particular pathway. To facilitate the exploration of the pathways, individuals were provided with a series of anonymized transcripts of therapeutic interactions that occurred in 2018. Depending on the pathway, the transcripts were either taken from sessions with professionals or from interactions on the online forum. To ensure confidentiality and privacy, the content of the transcripts was managed within the service evaluation limitations of the organization’s clinical audit guidelines.

Following a brief training session introducing qualitative research methods and the process of conducting thematic analysis [49], the following instructions were provided to each group of practitioner researchers:

- Inductively analyze a series of anonymized transcripts using the protocols of thematic analysis.
- Develop an overarching thematic map to provide a summary of the analysis in a common format of themes, subthemes, and codes.

Research supervision and advice were provided during this process. The findings of these exploratory studies were presented in a second workshop event and helped the working group understand how the different pathways worked and interacted. Following the presentations, all of the individuals who had been involved in examining the specific pathways worked collaboratively to articulate a ToC for the whole organization. This process involved deductively working to identify the agreed core elements of the ToC. These included the 3 overarching elements: (1) the *conditions* present for individuals wanting to access anonymous web-based therapy and support (including the CYP characteristics); (2) the mode of delivery (including the service inputs, worker activities, CYP activities, and the associated outputs); and (3) the reported and observed change (including the desired outcomes and the associated impact). The draft of this ToC was developed by an expert in the ToC methodology (JG) in collaboration with all other working group members.

### Phase 3: Coherence Checking the ToC

Once the draft ToC had been agreed upon by all members of the working group, the ToC was opened up for consultation with other core stakeholders of the Kooth community. This was a multifaceted strategy that sought comments about the coherence and plausibility of the ToC formulation of the overall presentation [50]. The wider stakeholder group that was consulted included 7 young people, 9 service managers, and 29 practitioners working for the Kooth service. All individuals were asked to review and comment on the draft ToC using a web-based questionnaire. The questionnaire specifically sought comments about the core elements of the ToC. Following the consultation process, the draft ToC was reviewed once again by the working group and revised to accommodate the learning from these new viewpoints. The ToC was broadly accepted by all members of the wider stakeholder group, but a number of minor changes were made to the language of the document to enhance the clarity of the theory presented.

### Ethical Considerations

The initial stages of this project were conducted as consultation exercises between academic partners and professionals involved in Kooth. The final phase of the work, which involved producing a public facing output, was approved by the University Research Ethics Committee of the fourth author (JP).

## Results

This project set out to develop a ToC for web-based therapy and support services, Kooth. A sustained period of reflexive exploration, which combined academic expertise and professional wisdom, led to the description of the ToC reported and discussed below. Textbox 1 provides a summary of the specific ToC that was arrived at following the three phases of reflection. The ToC includes elements common to ToC methodology (ie, focusing on the conditions present for individuals to use such a service, the mode of delivery, and the change that was reported or observed) and has been adjusted in areas to fit the needs of the Kooth service. For instance, the activities of Kooth workers and young service users have been separated to delineate the different ways in which people are involved in the change process.

**The theory of change for Kooth.**

**Young person characteristics**

- Want support then and there or quickly
- Do not have or want family and friends to turn to—may be in a marginalized group
- Curious, exploring, or looking for information and reassurance
- Unable or unwilling to access face-to-face services
- Comfortable with a preference for web-based communication
- Seeking a nonjudgmental space on the web
- Seeking a different connection with others

**Triage or decision to offer service**

- Delivery
  - Service inputs
    - Skilled and experienced professionals
    - Flexible access platform available out of hours, and written information or articles available 24/7
    - Robust clinical governance and risk managed through clinical oversight
  - Worker activities
    - Building an empathetic relationship
    - Drawing on professional understanding of child and adolescent developments
    - Assessing distress and risk and tailoring responses
    - Giving information and signposting
    - Cocreating goals and solutions with young people
    - Identifying what has helped before
    - Encouraging reflection and taking responsibility
    - Exploring the young person’s relationship and support systems
  - Young person activities
    - Offloading their worries
    - Opening up, articulating, and sharing their story
    - Learning about mental health so they can understand their experiences
    - Exploring their thoughts and feelings
    - Building a trusted connection with the worker
    - Identifying coping skills and testing approaches
  - Outputs
    - Feels heard and has feelings validated
    - Gets information
    - Changes perspective or sees new options
    - Has experienced opening up to someone and built a relationship with a professional
    - Takes ownership of an issue
    - Starts to engage or has information about face-to-face services
    - Builds connections and a safe online and offline community
- Change
  - Desired outcomes
    - Safer or crisis reduced
    - Able to reflect on thoughts, feelings, and perceptions
    - Able to consider future strategies
    - Greater self-awareness and emotional regulation
    - Acknowledges a reduction in stress
    - Achieves personal goals and recognizes progress made
    - Feels a sense of community
  - Impact
    - Better able to manage current and future situations
    - Is able to demonstrate ambition and hope for the future
    - Increased confidence, personal responsibility, and ability to make decisions
    - Sets personal goals for change
    - Is aware that ongoing support is available—is not alone
    - Has a positive experience of a web-based space

## Discussion

### Principal Findings

In the following sections, we reflect on the major elements of ToC in chronological order, notably the conditions that are present for CYP to access such support, the mode of delivery offered, and the change reported and observed. The presentation refers to the different elements of the ToC; however, due to restrictions of space, it is not possible to refer directly to each component in detail.

### The Conditions Present for CYP to Want to Access Web-Based Therapy and Support: CYP-Directed Mental Health and Well-being Support

Web-based therapy and support have become important resources for individuals who cannot access traditional face-to-face support [14]. This includes those who cannot physically access support and includes those that might struggle psychologically to access it too [51]. During the COVID-19 pandemic, web-based psychological support became an important resource for individuals within countries that were placed in enforced lockdown periods where self-distancing and self-isolation measures became commonplace. The ToC described here reflects some of these benefits and highlights that CYP may use these resources as points of informational and emotional support [15,16]. As a consequence, individuals may choose to access a combination of resources, with informative static webpages about particular issues, peer discussion forums, and professional support and guidance. The desire to obtain different types of support has previously been observed in the goals articulated by clients in web-based therapy [52] and the way that individuals use online forums [53]. Therefore, psychological services that offer anonymous therapy and support on the web need to be prepared to offer a variety of resources to accommodate the informational and emotional needs for which individuals access services.

Another area of development in this project was the view that anonymous web-based resources offer a novel means of CYP accessing and directing their support. Young users of web-based therapy have previously expressed the importance of not providing identifiable materials [27,54]. What is evident here however is that this stretches much further. Unlike face-to-face support services that describe themselves as child-centered [55] or prize shared decision making [56], anonymous web-based environments provide CYP with the opportunity to take ownership of their support packages and direct their engagement as a default. This position might be aligned to active client theories in therapy [57], with individuals being active both in directing the support they access and within the relationships with supporters themselves. As a consequence, they can be a means of leveling the power differentials between adult professionals and CYP accessing services. Such a position is likely to prove challenging for some professionals who might see their professional experience being undermined or not accounted for. As such, anonymous psychological support arguably recalibrates how professionals might define child-centered support by extending definitions to allow CYP to direct their support as a default position.

### The Mode of Delivery: A Positive Virtual Ecosystem

**Figure 2:**
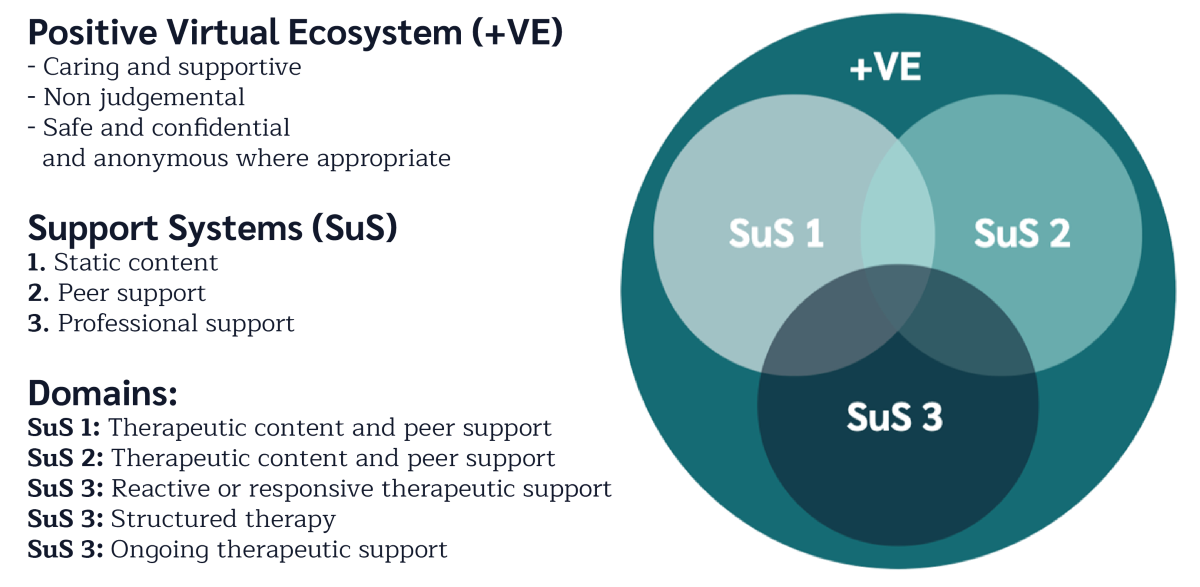
A positive virtual ecosystem (+VE).

In discussing and devising the service-level ToC, it was apparent that specific interventions are not offered in isolation. This may seem obvious, but many efficacy and effectiveness research designs often overlook the broader systems that impact the lives of individuals. Within the analysis process noted above, it was therefore apparent that the professionals developing the ToC were considering the way that individuals made use of a variety of resources on the Kooth website. For instance, the individuals discussed obtaining informational and emotional support from different pathways separately and in combination. As such, it may be possible to separate these elements into specific support systems in which individuals might access static support that does not change (eg, web-based content), peer support (eg, online forums), or professional support (eg, web-based therapy), but there was a general view that “the whole was greater than the sum of its parts,” with many reflecting that the young service users often evaluated their relationship with Kooth as a whole, rather than its specific components. As such, the Kooth service may be viewed as a broader ecosystem, involving a variety of resources that specifically aims to provide a safe and anonymous space for CYP in which they felt accepted to explore the issues that they encounter in life. The environment might therefore be viewed as akin to that advocated by person-centered therapists [22] or humanistic educationalists [58,59], in which individuals aim to develop a caring and supportive nonjudgmental environment for individuals to grow constructively. The Kooth service might therefore be viewed as a *positive virtual ecosystem* (+VE; Figure 2).

Psychological support services are relatively underdeveloped in terms of keeping up with technological advances [60]. Given this, it is common for services to attempt to replicate face-to-face ways of working when developing web-based therapeutic resources and to judge them using the same criteria [61]. The concept of +VE offers a more systemic perspective of how internet-based support might be more helpfully conceptualized, particularly given the current data-rich environments that are being created. Thus, rather than seeing specific interventions in isolation of one another, they might be viewed as part of a larger system in which all elements play an important role. Thus, if you take a section of the service away, such as the web-based static content, Kooth would have a different feel to it, and this would impact upon the other resources on offer.

Finally, when considering +VEs, the importance and value of static content and peer support needs to be acknowledged. These themselves can cater to individuals in need of informational support and, with regard to forums, emotional support from peers [62]. Given the large number of CYP who might benefit from additional support, and the limited finances often allocated to CYP services [10], appropriately curated content and moderated peer-support resources can be a positive way in which services might provide appropriate help to larger numbers of individuals.

### Understanding Change in Web-Based Therapy and Support for CYP

The desired outcomes noted in the ToC come as a consequence of young service users proactively engaging with the service inputs and activities of the professionals. They might be reduced to those activities that provide informational support (eg, giving information and signposting) and those that provide emotional support (eg, building an empathic relationship) [15]. These support types have already been well described within peer-support contexts on the web [62,63], and this frame can also be extended to describe the professional support offered. In accounting for this, when identifying whether CYP obtained what they wanted from the service, considering whether they received the information that they wanted and/or the engagement in supportive relationships proved important to consider.

Notably, the changes described in the ToC are broad and idiographic in nature. Furthermore, they did not reflect specific diagnostic criteria. As such, the changes identified might appear more aligned to the humanistic psychology perspective that underpins the service under scrutiny [20,23] and consolidates the view that traditional tools are unlikely to be relevant for all of those using these types of services [1]. In a similar way to the activities offered, one member of the working group (TH) suggested that the impact of the service might be reduced to a simplistic form. Specifically, they may be divided into those that have an impact on the intrapersonal world of the individuals (eg, able to reflect on thoughts, feelings, and perceptions) and those that impact their interpersonal worlds (eg, feeling a sense of community). This frame resonated strongly with other members of the working group. Considering the types of activities offered and the types of outcomes worked toward, a high-level outcome matrix might therefore be used to consider what a successful therapeutic engagement might look like for an anonymous web-based therapy and support service. Table 1 presents this matrix and provides an example of the types of response outcomes that CYP might articulate.

**Table 1:** High-level outcome matrix for Kooth services.

Outcome	Emotional support	Informational support
Intrapersonal	“I understand myself more”	“I can identify with something important to me”
Interpersonal	“It helps me relate to others”	“I have some skills I want to try with others”

### Implications for Professionals

The ToC that has been developed has numerous implications for mental health and well-being practitioners working in these environments. These include broad benefits, such as the ToC itself being an informative training tool for professionals working in this environment, to narrower elements, such as the need to develop bespoke tools to capture the outcomes of humanistic web-based support. These 2 areas were briefly considered.

It is important that professionals seriously consider the implications of transferring work to web-based formats. The ToC developed here helps to demonstrate the complexity inherent in working in this way. First, it highlights the need to be aware that some young people who access web-based support have different needs from their counterparts who access face-to-face support. Furthermore, the need for skilled and experienced professionals who are competent web-based communicators also comes to the fore. Although there are many transferable skills from face-to-face work, web-based resources, such as Kooth, are multifaceted and highlight the need for alternative or extended ways of thinking about support. In particular, it is important for professionals to consider the full offer on the web that is available to young people seeking help. The +VE that services provide can be adapted by service users in a multitude of ways. Such a position is in keeping with a pluralistic therapeutic approach that advocates that support should be led by the person seeking support [23] and that “one size can never fit all” [25]. Systemic thinking, which involves consideration of the different support systems being offered, therefore needs to be incorporated into the training of those supporting CYP on the web.

As previously indicated, capturing and measuring the change that individuals experience as a consequence of using a web-based counseling and support service proves to be challenging. Previous research has shown that standardized self-report measures developed for face-to-face support may not be transferable to web-based settings [1]. This seems particularly relevant when evaluating +VE. Given various ways in which individuals may tailor the support they are accessing, it is suggested that a flexible means of evaluating support is needed. As such, it is recommended that therapeutic outcomes are considered using self-report measures that make use of the outcome matrix previously noted. On the basis of this framework, an idiographic satisfaction measure can be used to complement other sources of personalized data collection, such as goal-based outcome measures.

### Strengths, Limitations, and Future Research

This is the first ToC to be created, focusing on a telepsychology resource for CYP. It provides a descriptive account of the types of people who might use these services, the resources needed to offer them, the activities that individuals engage in, and the outcomes and impacts that might be expected. The main strength of this conceptualization is its closeness to the organization that it reflects upon. Working alongside professionals from the organization to devise the project has helped to retain a direct currency for the partner organization. Such a position differs from other models that might adopt top-down approaches that ultimately lack ecological validity in application [38,39]. In contrast, it is acknowledged that this frame of understanding is underpinned by research groups’ underpinning in humanistic psychological principles [20,21]. As such, the synthesis of findings might be viewed as reflecting this more holistic positioning, and it is likely that others would see value in creating a more reductive frame that directly examines assessments focusing on pathological symptomology.

Going forward, it will be important to scrutinize the ideas presented in this paper in depth. In particular, we consider the following three areas to be core areas in need of further investigation:

- It is clear that anonymous web-based psychological support can prove to be liberating to some CYP. However, such freedom poses numerous challenges for professionals and services and therefore warrants further investigation. This can include reflecting upon practical issues, such as responding to risky behaviors, and relational issues, such as how disinhibition in web-based communication changes the therapeutic relationship.
- The +VE is currently unchartered territory. Numerous web-based services offer a variety of resources. In contrast to the creativity in the packages on offer, much evaluation remains focused on specific elements of the services (eg, web-based therapy). Given the data-rich nature of web-based resources, considering a more holistic picture becomes possible and analysis will arguably reflect the work of the services more fully. Furthermore, the systematic evaluation should not be limited to virtual ecosystems, and the CYP mental health and well-being support ecosystem should be extended to *in-person* work as well.
- Finally, a high-level outcome matrix needs to be considered alongside real-world activities. It will be possible to develop easy-to-complete satisfaction measures that are CYP friendly to capture information based on this framework. The utility of doing so however needs to be examined in depth.

### Conclusions

This project adds to the view that telepsychology directed toward CYP is diverse and complex to understand. This highlights the need to consider a broader virtual ecosystem and the interactions between different resources. More specifically, it describes the concept of the +VE in which a variety of interrelated resources are provided in a safe and caring overarching package. In doing so, it highlights the way in which services can support CYP to take ownership of and direct the support they obtain if they so desire. Such a position might be viewed as pluralistic in nature and differs greatly from the professionally led mental health and well-being services that are commonly offered face-to-face.

It is argued that web-based services available to CYP on the web should not solely aim to transplant face-to-face services on the web. Given the likelihood of telepsychology becoming more commonplace following the COVID-19 crisis, a variety of supportive approaches can be used to realize the full potential of the technology available. These resources can offer informational and emotional support in a variety of guises and include providing informative content, moderated online forums, stand-alone therapeutic programs, and professional psychological support such as therapy. By offering a variety of support options, individuals are able to tailor the support they access, and services can helpfully respond more fully to the differing needs and wants of the CYP seeking support.

Finally, the evaluation of the telepsychology activities offered on the web needs to be fit for purpose, with face-to-face resources arguably having limited transferability. The evaluation of interventions might focus on specific interventions, but by doing so, evaluation strategies can neglect the various ways in which individuals access and use web-based therapy and support services. Where resources are evaluated in a more holistic manner and include consideration of the broader virtual ecosystem, a higher level of assessment is needed to accommodate the complexity inherent in the variety of therapeutic resources on offer. Here, it is recommended that outcomes might be assessed around a matrix examining whether individuals received the informational or emotional support that they were seeking in conjunction with whether this was directed to supporting intrapersonal or interpersonal change. This simplistic frame might be presented in a palatable form so as to be appropriate for young individuals to use and be easily adopted in a web-based environment. Such a frame needs further exploration going forward but has scope to provide a useful means of assessing whether +VEs meet the specific needs of those accessing the services.

## Abbreviations

CAMHS: Child and Adolescent Mental Health Service
CYP: children and young people
ToC: theory of change
+VE: positive virtual ecosystem
